# Crystal structures of three *ortho*-substituted *N*-acyl­hydrazone derivatives

**DOI:** 10.1107/S2056989017016814

**Published:** 2017-11-28

**Authors:** H. Purandara, Sabine Foro, B. Thimme Gowda

**Affiliations:** a Department of Chemistry, Mangalore University, Mangalagangotri 574 199, Mangalore, India; b Department of Chemistry, Sri Dharmasthala Manjunatheshwara College (Autonomous), Ujire 574 240, India; c Institute of Materials Science, Darmstadt University of Technology, Alarich-Weiss-Strasse 2, D-64287, Darmstadt, Germany; d Karnataka State Rural Development and Panchayat Raj University, Gadag 582 101, India

**Keywords:** crystal structure, *N*-acyl­hydrazones, ring motifs, conformation, C—H⋯O inter­action

## Abstract

To explore the effect of the nature of substitutions on the structural parameters and hydrogen-bond inter­actions in *N*-acyl­hydrazone derivatives, the crystal structures of three *ortho*-substituted *N*-acyl­hydrazone derivatives, namely (*E*)-*N*-{2-[2-(2-chloro­benzyl­idene)hydrazin­yl]-2-oxoeth­yl}-4-methyl­benzene­sulfon­amide, C_16_H_16_ClN_3_O_3_S (I), (*E*)-*N*-{2-[2-(2-methyl­benzyl­idene)hydrazin­yl]-2-oxoeth­yl}-4-methyl­benzene­sulfonamide, C_17_H_19_N_3_O_3_S (II), and (*E*)-*N*-{2-[2-(2-nitro­benzyl­idene)hydrazin­yl]-2-oxoeth­yl}-4-methyl­benzene­sulfonamide, C_16_H_16_N_4_O_5_S (III), have been determined. The structures of the three compounds display similar mol­ecular conformations and hydrogen-bond patterns. The hydrazone part of the mol­ecule, C—C—N—N=C, is almost planar in all the compounds, with the C—C—N—N and C—N—N=C torsion angles being 179.5 (3) and 177.1 (3)°, respectively, in (I), −179.4 (2) and −177.1 (3)° in (II) and −179.7 (2) and 173.4 (2)° in (III). The two phenyl rings on either side of the chain are approximately parallel to each other. In the crystal, the mol­ecules are linked to each other *via* N—H⋯O hydrogen bonds, forming ribbons with *R*_2_^2^(8) and *R*_2_^2^(10) ring motifs. The introduction of electron-withdrawing groups (by a chloro or nitro group) to produce compounds (I) or (III) results in C—H⋯O hydrogen-bonding inter­actions involving the sulfonyl O atoms of adjacent ribbons, forming layers parallel to the *ab* plane in (I) or a three-dimensional network in (III). In (III), one O atom of the nitro group is disordered over two orientations with refined occupancy ratio of 0.836 (12):0.164 (12).

## Abstract 2

The effect of the nature of substitutions on the structural parameters and hydrogen-bonding inter­actions in *N*-acyl­hydrazone derivatives has been studied by synthesizing and determining the crystal structures of three *ortho*-substituted *N*-acyl­hydrazone derivatives, namely (*E*)-*N*-{2-[2-(2-chloro­benzyl­idene)hydrazin­yl]-2-oxoeth­yl}-4-methyl­benzene­sulfonamide (I), (*E*)-*N*-{2-[2-(2-methyl­benzyl­idene)hydrazin­yl]-2-oxoeth­yl}-4-methyl­benzene­sulfonamide (II) and (*E*)-*N*-{2-[2-(2-nitro­benzyl­idene)hydrazin­yl]-2-oxoeth­yl}-4-methyl­benzene­sulfonamide (III).

## Chemical context   

*N*-Acyl­hydrazones belong to the Schiff base family of general structure *R*_1_—C(=O)—N—N=C*R*_3_ *R*_4_. *N*-Acyl­hydrazones of aromatic aldehydes find great importance in organic synthesis due to their biological and medicinal activities (Tian *et al.*, 2009 ▸, 2011 ▸). The donor sites, carbonyl and imine groups, in the compounds are responsible for the physical and chemical properties of *N*-acyl­hydrazones. Their ability to form chelates with transition metals can be effectively utilized to analyse metals selectively as hydrazone complexes. *N*-Acyl­hydrazones can exist as *Z*/*E* geometrical isomers about the C=N bond of the hydrazone moiety (Palla *et al.*, 1986 ▸). Crystal-structure studies of *N*-acyl­hydrazones revealed that the mol­ecules display an *E* conformation in the solid state (Purandara *et al.*, 2015*a* ▸,*b* ▸,*c* ▸, 2017 ▸; Gu *et al.* 2012 ▸), whereas NMR spectroscopic studies showed the duplicate signals for amide and methyl­ene protons, indicating the presence of two isomers in solution (Lacerda *et al.*, 2012 ▸; Lopes *et al.*, 2013 ▸). As the stereochemistry of the hydrazone is determined by the various substituents in the hydrazone moiety, we thought it would be inter­esting to synthesize several *ortho-*substituted *N*-acyl­hydrazone derivatives to explore their effects on crystal-structure parameters and hydrogen-bonding inter­actions. Thus this paper describes the salient features of *ortho*-chloro-, methyl- and nitro-substituted *N*-acyl­hydrazone derivatives, namely, (*E*)-*N*-{2-[2-(2-chloro­benzyl­idene)hydrazin­yl]-2-oxoeth­yl}-4-methyl­benzene­sulfonamide, C_16_H_16_ClN_3_O_3_S (I), (*E*)-*N*-{2-[2-(2-methyl­benzyl­idene)hydrazin­yl]-2-oxoeth­yl}-4-methyl­benzene­sulfonamide, C_17_H_19_N_3_O_3_S (II), and (*E*)-*N*-{2-[2-(2-nitro­benzyl­idene)hydrazin­yl]-2-oxoeth­yl}-4-methyl­benzene­sulfonamide (III).
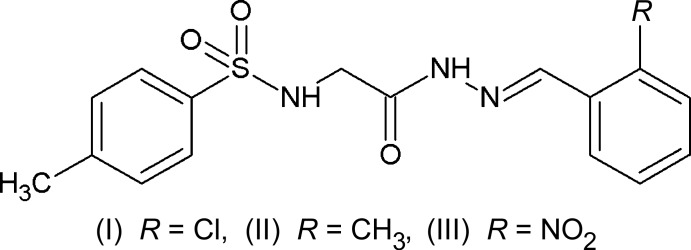


## Structural commentary   

**Figure 1:**
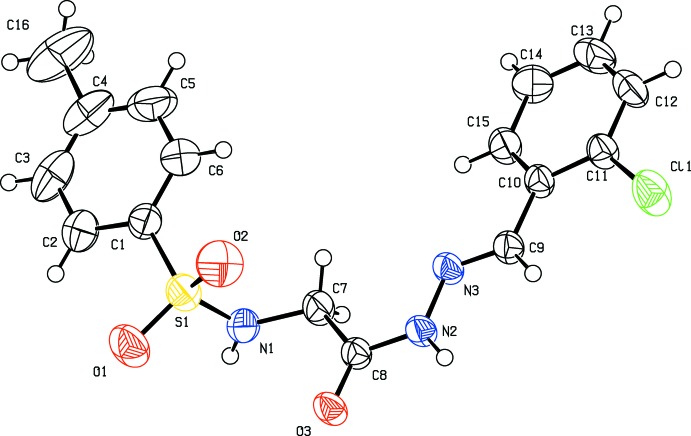
The mol­ecular structure of compound (I), with displacement ellipsoids drawn at the 50% probability level.

The title compounds (I)–(III) (Figs. 1 ▸–3 ▸ ▸), which differ only in the *ortho*-substituent, each crystallize in the centrosymmetric space group *P* 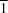 with one mol­ecule in the asymmetric units and display many common features. Each mol­ecule adopts an *E* configuration around the imine C=N bond. The conformation of the N—-H bond in the amide part is *syn* with respect to the C=O bond, the imine C—H bond and the *ortho* substituent. The sulfonamide bonds are found to be anti­clinal, and the torsion angles of the sulfonamide moieties are 98.6 (3), −99.6 (3) and 99.9 (2)° in compounds (I), (II), and (III), respectively.

The dihedral angles between the phenyl ring (C10-C15) and the mean plane of the C9/N3/N2/C8/O3 hydrazone fragment are 5.7 (2), 5.54 (18) and 7.90 (17)° for (I), (II), and (III), respectively. The *N*-acylhydrazone portion of the mol­ecules (C=N—NH—C=O group) is therefore approximately coplanar with the plane of benzyl­idenephenyl ring (C10–C15) in these compounds, but the sulfonyl glycine part of the mol­ecule is rotated by 40.0 (3)° in (I), 40.2 (3)° in (II) and 41.4 (2)° in (III) with respect to the hydrazone group. The phenyl rings are also approximately parallel to each other, forming dihedral angles ranging from 12.86 (11) to 13.10 (19)°. In (III), an intra­molecular C—H⋯O hydrogen bond involving the nitro group and the imine H atom is observed (Table 3 ▸).

## Supra­molecular features   

**Figure 4:**
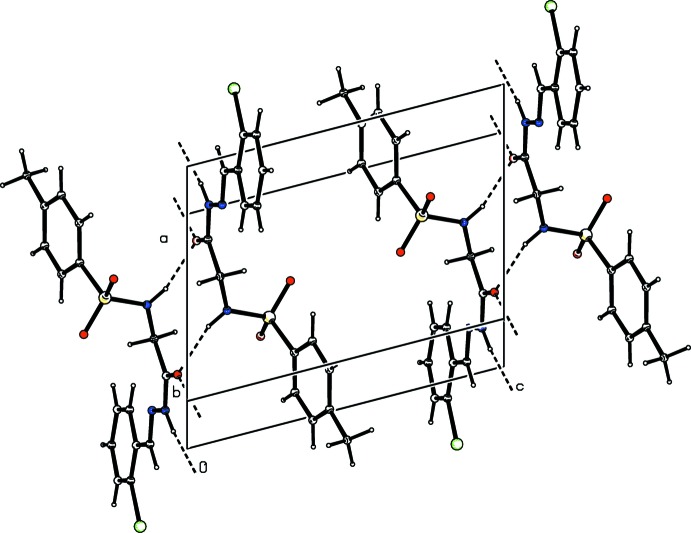
Crystal packing of compound (I), showing the formation of mol­ecular ribbons parallel to the *a* axis through N—H⋯O hydrogen bonds (dashed lines).

**Figure 5:**
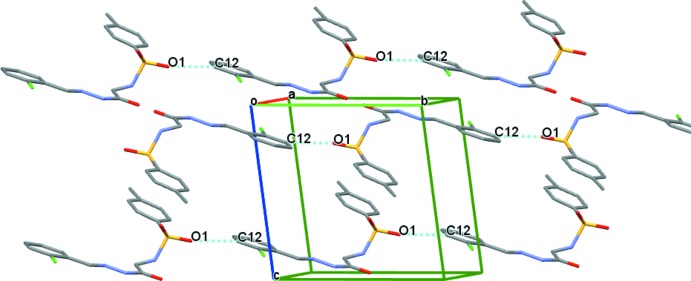
The C—H⋯O inter­actions (blue dotted lines) observed in the structure of the compound (I). H atoms have been omitted for clarity.

**Figure 6:**
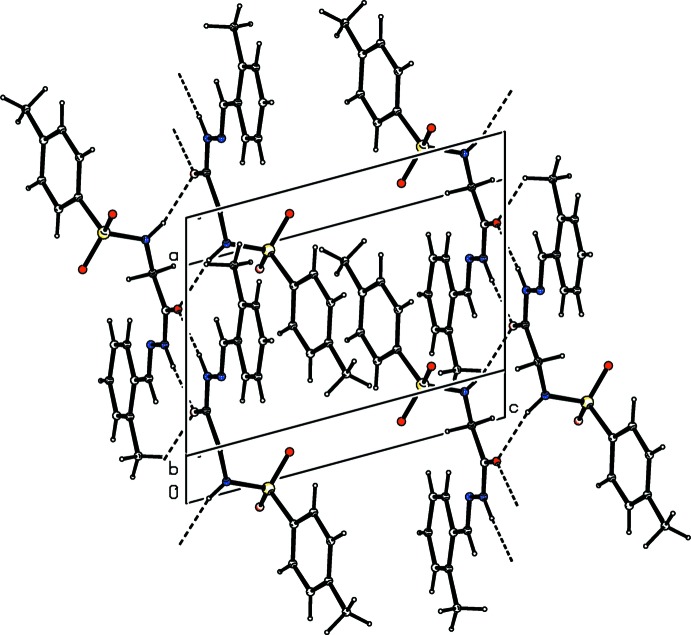
Crystal packing of compound (II), showing the formation of mol­ecular ribbons parallel to the *a* axis through N—H⋯O hydrogen bonds (dashed lines).

**Figure 7:**
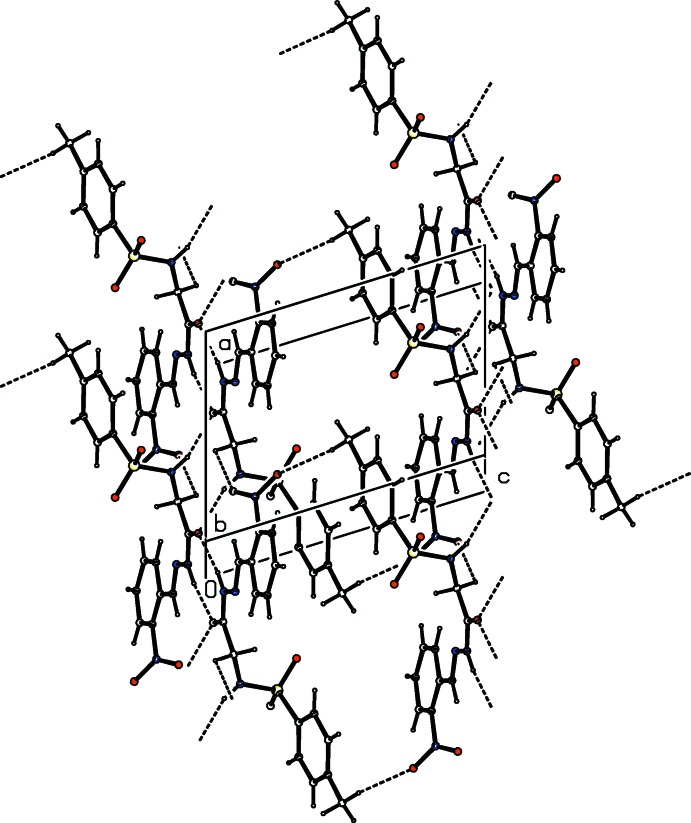
Crystal packing of compound (III), showing the formation of a three-dimensional network through N—H⋯O and C—H⋯O hydrogen bonds (dashed lines).

**Figure 8:**
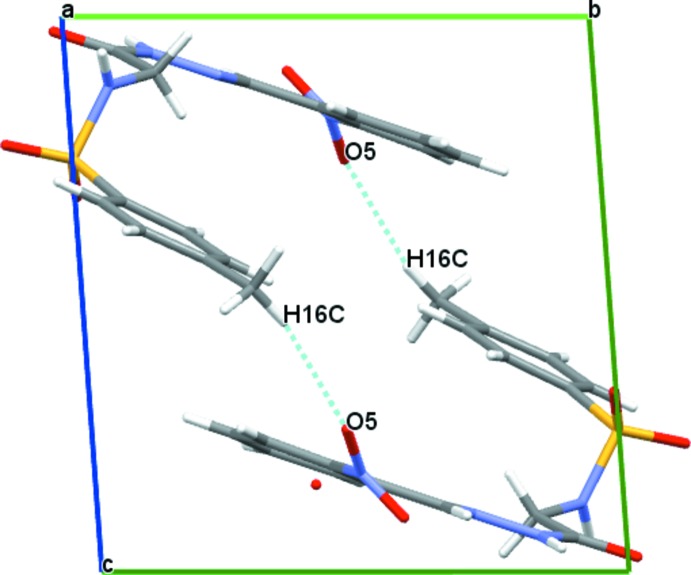
Partial crystal structure of compound (III), showing the C—H⋯O inter­action forming 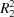
(34) rings along [001].

In all three compounds, the O atom of the carbonyl group is engaged as an acceptor in bifurcated N—H⋯O hydrogen bonding with the sulfonamide H atom and the amino H atom of the hydrazide segment of two centrosymmetrically related neighbouring mol­ecules, enclosing rings of 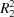
(8) and 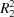
(10) graph-set motif and forming mol­ecular ribbons parallel to the *a* axis [Table 1 ▸, Fig. 4 ▸ for (I), Table 2 ▸, Fig. 6 ▸ for (II) and Table 3 ▸, Fig. 7 ▸ for (III)]. In the crystal structure of (II), there are no other significant inter­molecular inter­actions present. Replace­ment of the methyl group in (II) by the chloro or nitro electron-withdrawing groups to produce compound (I) or (III) introduces C—H⋯O inter­actions. In (I), the inter­actions involving the sulfonyl oxygen atoms and aromatic H atoms of adjacent ribbons (Fig. 5 ▸) result in the formation of two-dimensional layer networks extending parallel to the *ab* plane. In (III), the ribbons are further stabilized by inter­molecular C—H⋯O inter­actions between methyl­ene H atoms and the O4 oxygen atom of the nitro group. Adjacent ribbons in (III) are further linked into a three-dimensional network by weak hydrogen-bonding inter­actions occurring between methyl H atoms and the oxygen atom O5 of the nitro group, resulting in the formation of 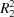
(34) ring motifs (Fig. 8 ▸).

## Database survey   

Comparison of structures (I)–(III) with those of the related *N*-acyl­hydrazone derivatives (*E*)-*N*-{2-[2-(4-methyl­benzyl­idene)hydrazin-1-yl]-2-oxoeth­yl}-*p*-toluene­sulfonamide (IV) (Purandara *et al.*, 2015*b* ▸) and (*E*)-*N*-2-[2-(4-nitro­benzyl­idene)hydrazine-1-yl]-2-oxoethyl-4-methyl­benzene­sulfon­amide *N*,*N*-di­methyl­formamide monosolvate (V) (Purandara *et al.*, 2017 ▸) indicate that mol­ecules of *ortho*-substituted compounds are U-shaped, while the mol­ecules of compounds (IV) and (V) have an extended chain conformation.

## Synthesis and crystallization   

**General procedure for the synthesis of** ***N*** **-(4-methyl­benzene­sulfon­yl)glycinyl hydrazide** (***L*** **3**)

*p*-Toluene­sulfonyl chloride (0.01 mol) was added to glycine (0.02 mol) dissolved in an aqueous solution of potassium carbonate (0.06 mol, 50 ml). The reaction mixture was stirred at 373 K for 6h, left overnight at room temperature, then filtered and treated with dilute hydro­chloric acid. The solid *N*-(4-methyl­benzene­sulfon­yl)glycine (*L*1) obtained was crystallized from aqueous ethanol. Sulfuric acid (0.5 ml) was added to (*L*1) (0.02 mol) dissolved in ethanol (30 ml) and the mixture was refluxed. The reaction mixture was monitored by TLC at regular inter­vals. After completion of the reaction, the reaction mixture was concentrated to remove excess ethanol. The product, *N*-(4-methyl­benzene­sulfon­yl)glycine ethyl ester (*L*2) was poured into water, neutralized with sodium bicarbonate and recrystallized from acetone. The pure (*L*2) (0.01 mol) was then added in small portions to a stirred solution of 99% hydrazine hydrate (10 ml) in 30 ml ethanol and the mixture was refluxed for 6 h. After cooling to room temperature, the resulting precipitate was filtered, washed with cold water and dried to obtain *N*-(4-methyl­benzene­sulfon­yl)glycinyl hydrazide (*L*3).

**Synthesis of compound (I)**

A mixture of *L*3 (0.01 mol) and 2-chloro­benzaldehyde (0.01 mol) in anhydrous methanol (30 ml) and two drops of glacial acetic acid was refluxed for 8 h. After cooling, the precipitate was collected by vacuum filtration, washed with cold methanol, dried, and recrystallized to a constant melting point from methanol (511–512 K). The purity of the compound was checked by TLC and characterized by its IR spectrum. The characteristic absorptions observed are 3199.9, 1674.2, 1604.8, 1327.0 and 1153.4 cm^−1^ for the asymmetric N—H, C=O, C=N, S=O and symmetric S=O stretching bands, respectively. ^1^H NMR (400 MHz, DMSO-*d*_6_, *δ* ppm): 2.36 (*s*, 3H), 3.56, 4.05 (*2d*, 2H, *J* = 6.1 Hz), 7.32–7.39 (*m*, 4H, Ar-H), 7.41–7.53 (*m*, 2H, Ar-H), 7.71–7.77 (*m*, 2H, Ar-H), 7.98 (*t*, 1H), 8.31, 8.57 (2*s*, 1H), 11.60 (*s*, 1H). ^13^C NMR (400 MHz, DMSO-*d*_6_, *δ* ppm): 20.96, 43.23, 44.63, 126.65, 127.38, 128.04, 129.70, 131.12, 132.99, 134.76, 137.50, 139.90, 142.50, 143.23, 158.17, 164.33, 169.08. Plate-shaped colourless single crystals of (I) suitable for the X-ray diffraction study were grown from a DMF solution by slow evaporation of the solvent.

S**ynthesis of compound (II)**

A mixture of *L*3 (0.01 mol) and 2-methyl­benzaldehyde (0.01 mol) in anhydrous methanol (30 ml) and two drops of glacial acetic acid was refluxed for 8 h. After cooling, the precipitate was collected by vacuum filtration, washed with cold methanol and dried. It was recrystallized to a constant melting point from methanol (474–475 K). The purity of the compound was checked by TLC and characterized by its IR spectrum. The characteristic absorptions observed are 3186.4, 1672.3, 1620.2, 1328.9 and 1155.4 cm^−1^ for the asymmetric N—H, C=O, C=N, S=O and symmetric S=O stretching bands, respectively. ^1^H NMR (400 MHz, DMSO-*d*_6_, *δ* ppm): 2.37 (*s*, 3H), 2.40 (*s*, 3H), 3.54, 4.01 (2*d*, 2H), 7.18–7.28 (*m*, 3H, Ar-H), 7.34 (*t*, 2H, Ar-H), 7.60–7.62 (*m*, 1H, Ar-H), 7.70–7.77 (*m*, 3H, Ar-H), 8.18, 8.46 (2*s*, 1H), 11.36 (*s*, 1H). ^13^C NMR (400 MHz, DMSO-*d*_6_, *δ* ppm): 19.53, 20.97, 43.33, 44.67, 125.93, 126.60, 129.34, 130.72, 131.86, 136.41, 136.67, 137.21, 137.61, 142.49, 143.07, 145.86, 163.98, 168.81. Prismatic colourless single crystals of (II) employed in the X-ray diffraction study were grown from a DMF solution by slow evaporation of the solvent.

S**ynthesis of compound (III)**

A mixture of *L*3 (0.01 mol) and 2-nitro­benzaldehyde (0.01 mol) in anhydrous methanol (30 ml) and two drops of glacial acetic acid was refluxed for 8 h. After cooling, the precipitate was collected by vacuum filtration, washed with cold methanol and dried. It was recrystallized to a constant melting point from methanol (509–512 K). The purity of the compound was checked by TLC and characterized by its IR spectrum. The characteristic absorptions observed are 3219.2, 1674.2, 1597.1, 1327.0 and 1132.2 cm^−1^ for the asymmetric N—H, C=O, C=N, S=O and symmetric S=O stretching bands, respectively. ^1^H NMR (400 MHz, DMSO-*d*_6_, *δ* ppm): 2.39 (*s*, 3H), 3.59, 4.04 (2*d*, 2H, *J* = 6.1 Hz), 7.35 (*t*, 2H, Ar-H), 7.60–7.66 (*m*, 1H, Ar-H), 7.73–7.77 (*m*, 4H, Ar-H), 7.95–8.06 (*m*, 2H, Ar-H), 8.33, 8.63 (2*s*, 1H), 11.72, 11.75 (2*s*, 1H). ^13^C NMR (400 MHz, DMSO-*d*_6_, *δ* ppm): 20.98, 43.22, 44.59, 124.38, 126.60, 128.07, 129.29, 130.29, 133.35, 137.22, 137.72, 139.12, 142.49, 147.86, 148.01, 164.54, 169.23. Rod-shaped light-yellow single crystals of (III) employed in the X-ray diffraction study were grown from a DMF solution by slow evaporation of the solvent.

## Refinement   

Crystal data, data collection and structure refinement details are summarized in Table 4 ▸. The amino H atoms were freely refined with the N—H distances restrained to 0.86 (2) Å. H atoms bonded to C were positioned with idealized geometry using a riding model with C—H = 0.93 Å (aromatic), 0.96 Å (meth­yl), 0.97 Å (methyl­ene). All H atoms were refined with isotropic displacement parameters set at 1.2*U*_eq_(C, N) or 1.5*U*_eq_(C) for methyl H atoms. A rotating model was used for the methyl groups. In the structure of (I), the *U*_ij_ components of atom C16 were restrained to approximate isotropic behavior. In (III), the O5 atom of the nitro group is disordered over two orientations with refined occupancy ratio of 0.836 (12):0.164 (12). The *U*_eq_ of atom O5′ was restrained to approximate isotropic behavior.

## Supplementary Material

Crystal structure: contains datablock(s) I, II, III. DOI: 10.1107/S2056989017016814/rz5225sup1.cif

Structure factors: contains datablock(s) I. DOI: 10.1107/S2056989017016814/rz5225Isup2.hkl

Structure factors: contains datablock(s) II. DOI: 10.1107/S2056989017016814/rz5225IIsup3.hkl

Structure factors: contains datablock(s) III. DOI: 10.1107/S2056989017016814/rz5225IIIsup4.hkl

Supporting information file. DOI: 10.1107/S2056989017016814/rz5225Isup5.cml

Supporting information file. DOI: 10.1107/S2056989017016814/rz5225IIsup6.cml

Supporting information file. DOI: 10.1107/S2056989017016814/rz5225IIIsup7.cml

CCDC references: 1433561, 1433612, 1433603

Additional supporting information:  crystallographic information; 3D view; checkCIF report

## Figures and Tables

**Figure 2:**
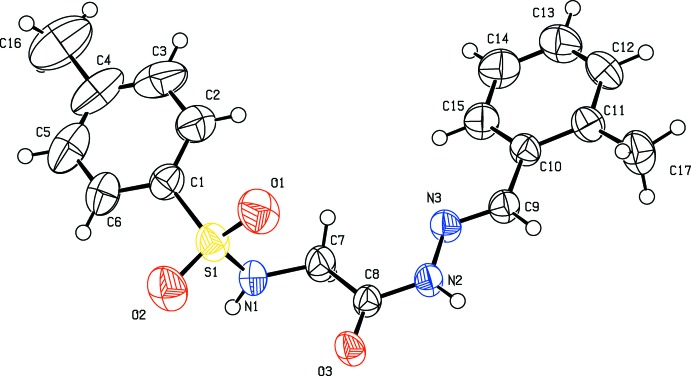
The mol­ecular structure of compound (II), with displacement ellipsoids drawn at the 50% probability level.

**Figure 3:**
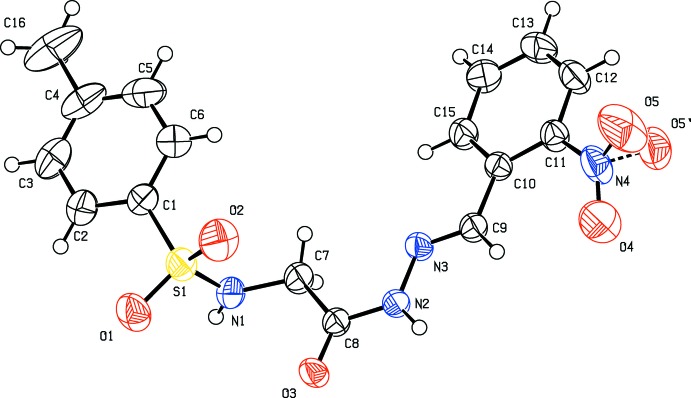
The mol­ecular structure of compound (III), with displacement ellipsoids drawn at the 50% probability level.

**Table 1:** Hydrogen-bond geometry (Å, °) for (I)

*D*—H⋯*A*	*D*—H	H⋯*A*	*D*⋯*A*	*D*—H⋯*A*
N1—H1*N*⋯O3^i^	0.84 (2)	2.02 (2)	2.839 (3)	162 (4)
N2—H2*N*⋯O3^ii^	0.86 (2)	2.03 (2)	2.884 (3)	171 (3)
C12—H12⋯O1^iii^	0.93	2.55	3.457 (4)	165

Symmetry codes: (i) 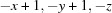; (ii) 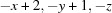; (iii) 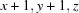.

**Table 2:** Hydrogen-bond geometry (Å, °) for (II)

*D*—H⋯*A*	*D*—H	H⋯*A*	*D*⋯*A*	*D*—H⋯*A*
N1—H1*N*⋯O3^i^	0.84 (2)	2.03 (2)	2.845 (3)	166 (3)
N2—H2*N*⋯O3^ii^	0.86 (2)	2.05 (2)	2.898 (3)	170 (3)

Symmetry codes: (i) 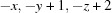; (ii) 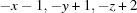.

**Table 3:** Hydrogen-bond geometry (Å, °) for (III)

*D*—H⋯*A*	*D*—H	H⋯*A*	*D*⋯*A*	*D*—H⋯*A*
N1—H1*N*⋯O3^i^	0.85 (2)	2.06 (2)	2.869 (3)	161 (3)
N2—H2*N*⋯O3^ii^	0.87 (2)	2.03 (2)	2.881 (3)	170 (3)
C7—H7*B*⋯O4^iii^	0.97	2.55	3.100 (4)	116
C9—H9*A*⋯O4	0.93	2.27	2.821 (4)	118
C16—H16*C*⋯O5^iv^	0.96	2.58	3.525 (6)	168

Symmetry codes: (i) 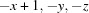; (ii) 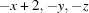; (iii) 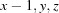; (iv) 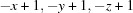.

**Table 4:** Experimental details

	(I)	(II)	(III)
Crystal data			
Chemical formula	C_16_H_16_ClN_3_O_3_S	C_17_H_19_N_3_O_3_S	C_16_H_16_N_4_O_5_S
*M*_r_	365.83	345.41	376.39
Crystal system, space group	Triclinic, *P* 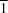	Triclinic, *P* 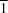	Triclinic, *P* 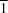
Temperature (K)	293	293	293
*a*, *b*, *c* (Å)	7.867 (1), 10.340 (1), 10.997 (2)	7.984 (1), 10.320 (2), 11.081 (2)	8.006 (1), 10.229 (1), 11.181 (2)
α, β, γ (°)	84.96 (1), 75.46 (1), 81.13 (1)	85.17 (1), 74.89 (1), 81.14 (1)	83.76 (1), 72.86 (1), 82.13 (1)
*V* (Å^3^)	854.4 (2)	870.0 (3)	864.5 (2)
*Z*	2	2	2
Radiation type	Mo *K*α	Mo *K*α	Mo *K*α
μ (mm^−1^)	0.37	0.21	0.22
Crystal size (mm)	0.50 × 0.36 × 0.18	0.30 × 0.16 × 0.12	0.48 × 0.48 × 0.28

Data collection			
Diffractometer	Oxford Diffraction Xcalibur diffractometer with Sapphire CCD detector	Oxford Diffraction Xcalibur diffractometer with Sapphire CCD detector	Oxford Diffraction Xcalibur diffractometer with Sapphire CCD detector
Absorption correction	Multi-scan *CrysAlis RED* (Oxford Diffraction, 2009 ▸)	Multi-scan *CrysAlis RED* (Oxford Diffraction, 2009 ▸)	Multi-scan *CrysAlis RED* (Oxford Diffraction, 2009 ▸)
*T*_min_, *T*_max_	0.838, 0.937	0.941, 0.976	0.900, 0.940
No. of measured, independent and observed [*I* > 2σ(*I*)] reflections	5855, 3444, 2709	5265, 3115, 2019	5980, 3524, 2511
*R*_int_	0.021	0.027	0.020
(sin θ/λ)_max_ (Å^−1^)	0.625	0.599	0.625

Refinement			
*R*[*F* ^2^ > 2σ(*F* ^2^)], *wR*(*F* ^2^), *S*	0.062, 0.131, 1.29	0.053, 0.141, 1.05	0.057, 0.131, 1.16
No. of reflections	3444	3115	3524
No. of parameters	224	225	252
No. of restraints	8	8	10
H-atom treatment	H atoms treated by a mixture of independent and constrained refinement	H atoms treated by a mixture of independent and constrained refinement	H atoms treated by a mixture of independent and constrained refinement
Δρ_max_, Δρ_min_ (e Å^−3^)	0.26, −0.28	0.28, −0.26	0.31, −0.30

Computer programs: *CrysAlis CCD* and *CrysAlis RED* (Oxford Diffraction, 2009 ▸), *SHELXS2013* (Sheldrick, 2008 ▸), *SHELXL2014* (Sheldrick, 2015 ▸) and *PLATON* (Spek, 2009 ▸).

## supplementary crystallographic information

### (E)-N-{2-[2-(2-Chlorobenzylidene)hydrazinyl]-2-oxoethyl}-4-methylbenzenesulfonamide (I) Crystal data

**Table:**

C_16_H_16_ClN_3_O_3_S	*Z* = 2
*M**_r_* = 365.83	*F*(000) = 380
Triclinic, *P*1	*D*_x_ = 1.422 Mg m^−^^3^
Hall symbol: -P 1	Mo *K*α radiation, λ = 0.71073 Å
*a* = 7.867 (1) Å	Cell parameters from 2213 reflections
*b* = 10.340 (1) Å	θ = 2.7–27.8°
*c* = 10.997 (2) Å	µ = 0.37 mm^−^^1^
α = 84.96 (1)°	*T* = 293 K
β = 75.46 (1)°	Plate, colourless
γ = 81.13 (1)°	0.50 × 0.36 × 0.18 mm
*V* = 854.4 (2) Å^3^	

### (E)-N-{2-[2-(2-Chlorobenzylidene)hydrazinyl]-2-oxoethyl}-4-methylbenzenesulfonamide (I) Data collection

**Table:**

Oxford Diffraction Xcalibur diffractometer with Sapphire CCD detector	2709 reflections with *I* > 2σ(*I*)
Radiation source: Enhance (Mo) X-ray Source	*R*_int_ = 0.021
Rotation method data acquisition using ω scans	θ_max_ = 26.4°, θ_min_ = 2.7°
Absorption correction: multi-scan CrysAlis RED (Oxford Diffraction, 2009)	*h* = −9→9
*T*_min_ = 0.838, *T*_max_ = 0.937	*k* = −12→12
5855 measured reflections	*l* = −13→13
3444 independent reflections	

### (E)-N-{2-[2-(2-Chlorobenzylidene)hydrazinyl]-2-oxoethyl}-4-methylbenzenesulfonamide (I) Refinement

**Table:**

Refinement on *F*^2^	8 restraints
Least-squares matrix: full	Hydrogen site location: mixed
*R*[*F*^2^ > 2σ(*F*^2^)] = 0.062	H atoms treated by a mixture of independent and constrained refinement
*wR*(*F*^2^) = 0.131	*w* = 1/[σ^2^(*F*_o_^2^) + (0.0196*P*)^2^ + 0.9514*P*] where *P* = (*F*_o_^2^ + 2*F*_c_^2^)/3
*S* = 1.29	(Δ/σ)_max_ = 0.001
3444 reflections	Δρ_max_ = 0.26 e Å^−^^3^
224 parameters	Δρ_min_ = −0.28 e Å^−^^3^

### (E)-N-{2-[2-(2-Chlorobenzylidene)hydrazinyl]-2-oxoethyl}-4-methylbenzenesulfonamide (I) Special details

**Table:**

Geometry. All esds (except the esd in the dihedral angle between two l.s. planes) are estimated using the full covariance matrix. The cell esds are taken into account individually in the estimation of esds in distances, angles and torsion angles; correlations between esds in cell parameters are only used when they are defined by crystal symmetry. An approximate (isotropic) treatment of cell esds is used for estimating esds involving l.s. planes.

### (E)-N-{2-[2-(2-Chlorobenzylidene)hydrazinyl]-2-oxoethyl}-4-methylbenzenesulfonamide (I) Fractional atomic coordinates and isotropic or equivalent isotropic displacement parameters (Å^2^)

**Table:**

	*x*	*y*	*z*	*U*_iso_*/*U*_eq_	
C1	0.1690 (4)	0.5986 (3)	0.3391 (3)	0.0455 (8)	
C2	0.0073 (5)	0.5621 (4)	0.3404 (3)	0.0553 (9)	
H2	0.0019	0.4856	0.3039	0.066*	
C3	−0.1476 (5)	0.6406 (5)	0.3968 (4)	0.0728 (13)	
H3	−0.2567	0.6152	0.3986	0.087*	
C4	−0.1428 (6)	0.7543 (5)	0.4497 (4)	0.0762 (13)	
C5	0.0198 (7)	0.7875 (4)	0.4496 (4)	0.0803 (14)	
H5	0.0250	0.8637	0.4867	0.096*	
C6	0.1762 (6)	0.7103 (4)	0.3955 (4)	0.0655 (11)	
H6	0.2849	0.7338	0.3974	0.079*	
C7	0.5616 (4)	0.6710 (3)	0.1041 (4)	0.0472 (8)	
H7A	0.5499	0.7162	0.1800	0.057*	
H7B	0.5426	0.7365	0.0384	0.057*	
C8	0.7477 (4)	0.5978 (3)	0.0656 (3)	0.0363 (7)	
C9	0.9704 (4)	0.8454 (3)	0.1176 (3)	0.0384 (7)	
H9	1.0861	0.8028	0.0963	0.046*	
C10	0.9332 (4)	0.9799 (3)	0.1602 (3)	0.0376 (7)	
C11	1.0632 (4)	1.0477 (3)	0.1782 (3)	0.0416 (7)	
C12	1.0240 (5)	1.1749 (3)	0.2181 (4)	0.0541 (9)	
H12	1.1130	1.2182	0.2307	0.065*	
C13	0.8524 (5)	1.2361 (3)	0.2387 (4)	0.0595 (10)	
H13	0.8251	1.3213	0.2655	0.071*	
C14	0.7207 (5)	1.1729 (3)	0.2202 (4)	0.0588 (10)	
H14	0.6049	1.2154	0.2336	0.071*	
C15	0.7608 (4)	1.0463 (3)	0.1817 (3)	0.0483 (8)	
H15	0.6706	1.0040	0.1698	0.058*	
C16	−0.3116 (7)	0.8436 (6)	0.5055 (5)	0.126 (2)	
H16A	−0.4108	0.7957	0.5215	0.188*	
H16B	−0.3280	0.9166	0.4473	0.188*	
H16C	−0.3026	0.8751	0.5829	0.188*	
N1	0.4270 (4)	0.5847 (3)	0.1266 (3)	0.0500 (7)	
H1N	0.381 (5)	0.572 (4)	0.068 (3)	0.060*	
N2	0.8803 (3)	0.6631 (2)	0.0693 (3)	0.0388 (6)	
H2N	0.987 (3)	0.625 (3)	0.044 (3)	0.047*	
N3	0.8414 (3)	0.7884 (2)	0.1104 (2)	0.0386 (6)	
O1	0.3203 (3)	0.3846 (2)	0.2318 (3)	0.0677 (8)	
O2	0.4985 (3)	0.5068 (3)	0.3261 (3)	0.0708 (8)	
O3	0.7779 (3)	0.4849 (2)	0.0302 (2)	0.0445 (6)	
Cl1	1.28438 (12)	0.97633 (10)	0.14816 (11)	0.0677 (3)	
S1	0.36582 (11)	0.50602 (8)	0.25882 (9)	0.0486 (2)	

### (E)-N-{2-[2-(2-Chlorobenzylidene)hydrazinyl]-2-oxoethyl}-4-methylbenzenesulfonamide (I) Atomic displacement parameters (Å^2^)

**Table:**

	*U*^11^	*U*^22^	*U*^33^	*U*^12^	*U*^13^	*U*^23^
C1	0.0432 (18)	0.0427 (18)	0.0470 (19)	−0.0067 (14)	−0.0047 (15)	−0.0005 (15)
C2	0.047 (2)	0.061 (2)	0.058 (2)	−0.0089 (17)	−0.0117 (17)	0.0025 (18)
C3	0.041 (2)	0.107 (4)	0.059 (3)	−0.002 (2)	−0.0046 (18)	0.015 (3)
C4	0.070 (3)	0.084 (3)	0.052 (2)	0.018 (2)	0.006 (2)	0.012 (2)
C5	0.101 (4)	0.060 (3)	0.063 (3)	0.001 (2)	0.008 (3)	−0.018 (2)
C6	0.065 (2)	0.063 (3)	0.065 (3)	−0.013 (2)	−0.001 (2)	−0.017 (2)
C7	0.0312 (16)	0.0410 (18)	0.067 (2)	−0.0049 (13)	−0.0036 (15)	−0.0137 (16)
C8	0.0335 (15)	0.0321 (16)	0.0429 (17)	−0.0045 (12)	−0.0077 (13)	−0.0036 (13)
C9	0.0335 (15)	0.0310 (15)	0.0487 (19)	−0.0035 (12)	−0.0062 (14)	−0.0036 (14)
C10	0.0371 (16)	0.0293 (15)	0.0456 (18)	−0.0065 (12)	−0.0080 (14)	−0.0005 (13)
C11	0.0409 (17)	0.0337 (16)	0.0515 (19)	−0.0070 (13)	−0.0125 (14)	−0.0014 (14)
C12	0.067 (2)	0.0386 (19)	0.063 (2)	−0.0193 (17)	−0.0191 (19)	−0.0059 (17)
C13	0.071 (3)	0.0337 (18)	0.073 (3)	−0.0028 (17)	−0.015 (2)	−0.0138 (18)
C14	0.052 (2)	0.041 (2)	0.076 (3)	0.0058 (16)	−0.0056 (19)	−0.0110 (18)
C15	0.0385 (17)	0.0392 (18)	0.066 (2)	−0.0064 (14)	−0.0073 (16)	−0.0083 (16)
C16	0.101 (4)	0.134 (5)	0.090 (4)	0.060 (4)	0.022 (3)	0.008 (3)
N1	0.0338 (14)	0.0640 (19)	0.0539 (18)	−0.0144 (13)	−0.0039 (13)	−0.0180 (15)
N2	0.0297 (13)	0.0296 (13)	0.0553 (17)	−0.0004 (10)	−0.0059 (12)	−0.0115 (12)
N3	0.0374 (14)	0.0272 (13)	0.0499 (16)	−0.0041 (10)	−0.0069 (12)	−0.0058 (11)
O1	0.0643 (17)	0.0380 (13)	0.100 (2)	−0.0060 (12)	−0.0151 (15)	−0.0128 (14)
O2	0.0538 (16)	0.083 (2)	0.081 (2)	0.0014 (14)	−0.0306 (14)	−0.0075 (16)
O3	0.0352 (11)	0.0325 (12)	0.0662 (15)	−0.0013 (9)	−0.0107 (10)	−0.0148 (11)
Cl1	0.0408 (5)	0.0574 (6)	0.1094 (9)	−0.0058 (4)	−0.0234 (5)	−0.0156 (5)
S1	0.0380 (4)	0.0432 (5)	0.0646 (6)	−0.0030 (3)	−0.0122 (4)	−0.0084 (4)

### (E)-N-{2-[2-(2-Chlorobenzylidene)hydrazinyl]-2-oxoethyl}-4-methylbenzenesulfonamide (I) Geometric parameters (Å, º)

**Table:**

C1—C6	1.373 (5)	C10—C11	1.387 (4)
C1—C2	1.378 (5)	C10—C15	1.396 (4)
C1—S1	1.760 (3)	C11—C12	1.388 (4)
C2—C3	1.390 (5)	C11—Cl1	1.744 (3)
C2—H2	0.9300	C12—C13	1.372 (5)
C3—C4	1.366 (7)	C12—H12	0.9300
C3—H3	0.9300	C13—C14	1.370 (5)
C4—C5	1.375 (7)	C13—H13	0.9300
C4—C16	1.518 (6)	C14—C15	1.377 (5)
C5—C6	1.385 (6)	C14—H14	0.9300
C5—H5	0.9300	C15—H15	0.9300
C6—H6	0.9300	C16—H16A	0.9600
C7—N1	1.449 (4)	C16—H16B	0.9600
C7—C8	1.517 (4)	C16—H16C	0.9600
C7—H7A	0.9700	N1—S1	1.603 (3)
C7—H7B	0.9700	N1—H1N	0.843 (18)
C8—O3	1.232 (3)	N2—N3	1.374 (3)
C8—N2	1.338 (4)	N2—H2N	0.863 (18)
C9—N3	1.272 (4)	O1—S1	1.432 (3)
C9—C10	1.469 (4)	O2—S1	1.424 (3)
C9—H9	0.9300		
			
C6—C1—C2	120.0 (3)	C10—C11—Cl1	120.7 (2)
C6—C1—S1	120.1 (3)	C12—C11—Cl1	117.4 (3)
C2—C1—S1	119.9 (3)	C13—C12—C11	119.1 (3)
C1—C2—C3	119.5 (4)	C13—C12—H12	120.4
C1—C2—H2	120.3	C11—C12—H12	120.4
C3—C2—H2	120.3	C14—C13—C12	120.7 (3)
C4—C3—C2	121.3 (4)	C14—C13—H13	119.6
C4—C3—H3	119.4	C12—C13—H13	119.6
C2—C3—H3	119.4	C13—C14—C15	119.7 (3)
C3—C4—C5	118.3 (4)	C13—C14—H14	120.2
C3—C4—C16	121.4 (5)	C15—C14—H14	120.2
C5—C4—C16	120.3 (5)	C14—C15—C10	121.7 (3)
C4—C5—C6	121.6 (4)	C14—C15—H15	119.2
C4—C5—H5	119.2	C10—C15—H15	119.2
C6—C5—H5	119.2	C4—C16—H16A	109.5
C1—C6—C5	119.3 (4)	C4—C16—H16B	109.5
C1—C6—H6	120.4	H16A—C16—H16B	109.5
C5—C6—H6	120.4	C4—C16—H16C	109.5
N1—C7—C8	112.3 (3)	H16A—C16—H16C	109.5
N1—C7—H7A	109.1	H16B—C16—H16C	109.5
C8—C7—H7A	109.1	C7—N1—S1	122.4 (3)
N1—C7—H7B	109.1	C7—N1—H1N	120 (3)
C8—C7—H7B	109.1	S1—N1—H1N	118 (3)
H7A—C7—H7B	107.9	C8—N2—N3	119.2 (2)
O3—C8—N2	120.9 (3)	C8—N2—H2N	118 (2)
O3—C8—C7	122.6 (3)	N3—N2—H2N	123 (2)
N2—C8—C7	116.5 (3)	C9—N3—N2	117.4 (2)
N3—C9—C10	118.7 (3)	O2—S1—O1	120.41 (17)
N3—C9—H9	120.6	O2—S1—N1	107.22 (16)
C10—C9—H9	120.6	O1—S1—N1	106.85 (17)
C11—C10—C15	116.9 (3)	O2—S1—C1	108.52 (17)
C11—C10—C9	123.2 (3)	O1—S1—C1	107.29 (16)
C15—C10—C9	119.9 (3)	N1—S1—C1	105.64 (16)
C10—C11—C12	121.9 (3)		
			
C6—C1—C2—C3	1.4 (6)	C11—C12—C13—C14	0.0 (6)
S1—C1—C2—C3	−175.7 (3)	C12—C13—C14—C15	0.6 (6)
C1—C2—C3—C4	0.8 (6)	C13—C14—C15—C10	−0.3 (6)
C2—C3—C4—C5	−2.1 (6)	C11—C10—C15—C14	−0.6 (5)
C2—C3—C4—C16	176.9 (4)	C9—C10—C15—C14	−179.2 (3)
C3—C4—C5—C6	1.3 (7)	C8—C7—N1—S1	84.5 (3)
C16—C4—C5—C6	−177.7 (4)	O3—C8—N2—N3	−179.4 (3)
C2—C1—C6—C5	−2.2 (6)	C7—C8—N2—N3	2.0 (4)
S1—C1—C6—C5	174.9 (3)	C10—C9—N3—N2	179.5 (3)
C4—C5—C6—C1	0.9 (7)	C8—N2—N3—C9	177.1 (3)
N1—C7—C8—O3	15.4 (5)	C7—N1—S1—O2	−17.0 (3)
N1—C7—C8—N2	−166.0 (3)	C7—N1—S1—O1	−147.3 (3)
N3—C9—C10—C11	176.2 (3)	C7—N1—S1—C1	98.6 (3)
N3—C9—C10—C15	−5.3 (5)	C6—C1—S1—O2	37.5 (4)
C15—C10—C11—C12	1.2 (5)	C2—C1—S1—O2	−145.4 (3)
C9—C10—C11—C12	179.8 (3)	C6—C1—S1—O1	169.0 (3)
C15—C10—C11—Cl1	−177.5 (3)	C2—C1—S1—O1	−13.8 (3)
C9—C10—C11—Cl1	1.1 (5)	C6—C1—S1—N1	−77.2 (3)
C10—C11—C12—C13	−0.9 (5)	C2—C1—S1—N1	99.9 (3)
Cl1—C11—C12—C13	177.8 (3)		

### (E)-N-{2-[2-(2-Chlorobenzylidene)hydrazinyl]-2-oxoethyl}-4-methylbenzenesulfonamide (I) Hydrogen-bond geometry (Å, º)

**Table:**

*D*—H···*A*	*D*—H	H···*A*	*D*···*A*	*D*—H···*A*
N1—H1*N*···O3^i^	0.84 (2)	2.02 (2)	2.839 (3)	162 (4)
N2—H2*N*···O3^ii^	0.86 (2)	2.03 (2)	2.884 (3)	171 (3)
C12—H12···O1^iii^	0.93	2.55	3.457 (4)	165

Symmetry codes: (i) −*x*+1, −*y*+1, −*z*; (ii) −*x*+2, −*y*+1, −*z*; (iii) *x*+1, *y*+1, *z*.

### (E)-N-{2-[2-(2-Methylbenzylidene)hydrazinyl]-2-oxoethyl}-4-methylbenzenesulfonamide (II) Crystal data

**Table:**

C_17_H_19_N_3_O_3_S	*Z* = 2
*M**_r_* = 345.41	*F*(000) = 364
Triclinic, *P*1	*D*_x_ = 1.319 Mg m^−^^3^
*a* = 7.984 (1) Å	Mo *K*α radiation, λ = 0.71073 Å
*b* = 10.320 (2) Å	Cell parameters from 2050 reflections
*c* = 11.081 (2) Å	θ = 2.7–27.9°
α = 85.17 (1)°	µ = 0.21 mm^−^^1^
β = 74.89 (1)°	*T* = 293 K
γ = 81.14 (1)°	Prism, colourless
*V* = 870.0 (3) Å^3^	0.30 × 0.16 × 0.12 mm

### (E)-N-{2-[2-(2-Methylbenzylidene)hydrazinyl]-2-oxoethyl}-4-methylbenzenesulfonamide (II) Data collection

**Table:**

Oxford Diffraction Xcalibur diffractometer with Sapphire CCD detector	2019 reflections with *I* > 2σ(*I*)
Radiation source: Enhance (Mo) X-ray Source	*R*_int_ = 0.027
Rotation method data acquisition using ω scans	θ_max_ = 25.2°, θ_min_ = 2.7°
Absorption correction: multi-scan CrysAlis RED (Oxford Diffraction, 2009)	*h* = −5→9
*T*_min_ = 0.941, *T*_max_ = 0.976	*k* = −12→12
5265 measured reflections	*l* = −13→13
3115 independent reflections	

### (E)-N-{2-[2-(2-Methylbenzylidene)hydrazinyl]-2-oxoethyl}-4-methylbenzenesulfonamide (II) Refinement

**Table:**

Refinement on *F*^2^	8 restraints
Least-squares matrix: full	Hydrogen site location: mixed
*R*[*F*^2^ > 2σ(*F*^2^)] = 0.053	H atoms treated by a mixture of independent and constrained refinement
*wR*(*F*^2^) = 0.141	*w* = 1/[σ^2^(*F*_o_^2^) + (0.0571*P*)^2^ + 0.4335*P*] where *P* = (*F*_o_^2^ + 2*F*_c_^2^)/3
*S* = 1.05	(Δ/σ)_max_ = 0.006
3115 reflections	Δρ_max_ = 0.28 e Å^−^^3^
225 parameters	Δρ_min_ = −0.26 e Å^−^^3^

### (E)-N-{2-[2-(2-Methylbenzylidene)hydrazinyl]-2-oxoethyl}-4-methylbenzenesulfonamide (II) Special details

**Table:**

Geometry. All esds (except the esd in the dihedral angle between two l.s. planes) are estimated using the full covariance matrix. The cell esds are taken into account individually in the estimation of esds in distances, angles and torsion angles; correlations between esds in cell parameters are only used when they are defined by crystal symmetry. An approximate (isotropic) treatment of cell esds is used for estimating esds involving l.s. planes.

### (E)-N-{2-[2-(2-Methylbenzylidene)hydrazinyl]-2-oxoethyl}-4-methylbenzenesulfonamide (II) Fractional atomic coordinates and isotropic or equivalent isotropic displacement parameters (Å^2^)

**Table:**

	*x*	*y*	*z*	*U*_iso_*/*U*_eq_	
C1	0.3267 (4)	0.4077 (3)	0.6615 (3)	0.0531 (8)	
C2	0.3217 (5)	0.2980 (4)	0.6002 (4)	0.0746 (10)	
H2	0.2153	0.2759	0.5950	0.089*	
C3	0.4765 (7)	0.2216 (4)	0.5467 (4)	0.0934 (13)	
H3	0.4733	0.1477	0.5052	0.112*	
C4	0.6359 (6)	0.2521 (5)	0.5531 (4)	0.0929 (14)	
C5	0.6381 (5)	0.3645 (5)	0.6099 (4)	0.0863 (13)	
H5	0.7449	0.3893	0.6108	0.104*	
C6	0.4851 (4)	0.4414 (4)	0.6656 (3)	0.0652 (9)	
H6	0.4889	0.5160	0.7059	0.078*	
C7	−0.0626 (3)	0.3333 (3)	0.8958 (3)	0.0552 (8)	
H7A	−0.0428	0.2674	0.9601	0.066*	
H7B	−0.0510	0.2884	0.8200	0.066*	
C8	−0.2477 (3)	0.4048 (3)	0.9366 (3)	0.0410 (6)	
C9	−0.4635 (4)	0.1560 (3)	0.8824 (3)	0.0458 (7)	
H9	−0.5775	0.1991	0.9048	0.055*	
C10	−0.4289 (4)	0.0216 (3)	0.8385 (3)	0.0441 (7)	
C11	−0.5630 (4)	−0.0429 (3)	0.8232 (3)	0.0504 (7)	
C12	−0.5183 (5)	−0.1718 (3)	0.7823 (3)	0.0635 (9)	
H12	−0.6054	−0.2162	0.7707	0.076*	
C13	−0.3499 (5)	−0.2332 (3)	0.7591 (4)	0.0707 (10)	
H13	−0.3242	−0.3186	0.7321	0.085*	
C14	−0.2182 (5)	−0.1709 (3)	0.7750 (4)	0.0704 (10)	
H14	−0.1039	−0.2137	0.7599	0.084*	
C15	−0.2575 (4)	−0.0438 (3)	0.8136 (3)	0.0576 (8)	
H15	−0.1681	−0.0007	0.8232	0.069*	
C16	0.8040 (7)	0.1637 (5)	0.4972 (5)	0.1340 (19)	
H16A	0.7763	0.0839	0.4733	0.201*	
H16B	0.8717	0.1437	0.5580	0.201*	
H16C	0.8704	0.2078	0.4248	0.201*	
C17	−0.7500 (4)	0.0184 (3)	0.8499 (4)	0.0708 (10)	
H17A	−0.8199	−0.0420	0.8323	0.106*	
H17B	−0.7602	0.0972	0.7983	0.106*	
H17C	−0.7901	0.0393	0.9364	0.106*	
N1	0.0695 (3)	0.4192 (3)	0.8728 (3)	0.0584 (7)	
H1N	0.117 (4)	0.434 (3)	0.928 (3)	0.070*	
N2	−0.3764 (3)	0.3385 (2)	0.9324 (2)	0.0452 (6)	
H2N	−0.484 (2)	0.373 (3)	0.959 (3)	0.054*	
N3	−0.3362 (3)	0.2126 (2)	0.8894 (2)	0.0448 (6)	
O1	0.0009 (3)	0.4970 (3)	0.6754 (3)	0.0814 (8)	
O2	0.1736 (3)	0.6208 (2)	0.7693 (3)	0.0779 (8)	
O3	−0.2783 (2)	0.51737 (17)	0.9738 (2)	0.0505 (5)	
S1	0.13061 (10)	0.49880 (8)	0.74192 (9)	0.0576 (3)	

### (E)-N-{2-[2-(2-Methylbenzylidene)hydrazinyl]-2-oxoethyl}-4-methylbenzenesulfonamide (II) Atomic displacement parameters (Å^2^)

**Table:**

	*U*^11^	*U*^22^	*U*^33^	*U*^12^	*U*^13^	*U*^23^
C1	0.0511 (18)	0.0503 (18)	0.0533 (19)	−0.0046 (14)	−0.0075 (14)	0.0021 (15)
C2	0.073 (2)	0.072 (2)	0.073 (3)	−0.0126 (19)	−0.0026 (19)	−0.015 (2)
C3	0.120 (4)	0.067 (3)	0.070 (3)	0.006 (2)	0.008 (3)	−0.013 (2)
C4	0.079 (3)	0.106 (4)	0.060 (3)	0.028 (3)	0.012 (2)	0.021 (2)
C5	0.054 (2)	0.119 (4)	0.073 (3)	0.000 (2)	−0.0066 (19)	0.022 (3)
C6	0.0460 (19)	0.075 (2)	0.071 (2)	−0.0074 (16)	−0.0118 (16)	0.0067 (18)
C7	0.0345 (15)	0.0471 (17)	0.081 (2)	−0.0023 (12)	−0.0066 (14)	−0.0174 (16)
C8	0.0323 (14)	0.0387 (16)	0.0503 (17)	−0.0039 (12)	−0.0074 (12)	−0.0036 (13)
C9	0.0381 (15)	0.0389 (16)	0.0580 (19)	−0.0016 (12)	−0.0089 (13)	−0.0054 (13)
C10	0.0470 (16)	0.0340 (15)	0.0510 (18)	−0.0087 (12)	−0.0103 (13)	−0.0015 (13)
C11	0.0549 (18)	0.0410 (16)	0.0568 (19)	−0.0098 (13)	−0.0162 (15)	0.0008 (14)
C12	0.076 (2)	0.0443 (18)	0.077 (2)	−0.0181 (16)	−0.0252 (19)	−0.0066 (16)
C13	0.082 (3)	0.0419 (19)	0.087 (3)	−0.0032 (17)	−0.020 (2)	−0.0126 (18)
C14	0.063 (2)	0.050 (2)	0.089 (3)	0.0090 (16)	−0.0111 (19)	−0.0132 (19)
C15	0.0499 (18)	0.0430 (17)	0.075 (2)	−0.0011 (14)	−0.0085 (16)	−0.0098 (16)
C16	0.118 (3)	0.132 (3)	0.113 (3)	0.043 (3)	0.002 (2)	0.004 (3)
C17	0.054 (2)	0.067 (2)	0.097 (3)	−0.0127 (16)	−0.0249 (19)	−0.006 (2)
N1	0.0384 (14)	0.0756 (18)	0.0637 (19)	−0.0194 (12)	−0.0066 (12)	−0.0165 (15)
N2	0.0315 (12)	0.0340 (12)	0.0678 (17)	−0.0010 (10)	−0.0076 (11)	−0.0119 (11)
N3	0.0409 (13)	0.0310 (12)	0.0610 (16)	−0.0019 (10)	−0.0103 (11)	−0.0084 (11)
O1	0.0602 (15)	0.0930 (19)	0.099 (2)	0.0009 (13)	−0.0384 (14)	−0.0123 (16)
O2	0.0739 (16)	0.0413 (13)	0.119 (2)	−0.0044 (11)	−0.0254 (15)	−0.0112 (13)
O3	0.0393 (11)	0.0349 (11)	0.0787 (15)	−0.0017 (8)	−0.0153 (10)	−0.0159 (10)
S1	0.0435 (4)	0.0522 (5)	0.0781 (6)	−0.0020 (3)	−0.0178 (4)	−0.0089 (4)

### (E)-N-{2-[2-(2-Methylbenzylidene)hydrazinyl]-2-oxoethyl}-4-methylbenzenesulfonamide (II) Geometric parameters (Å, º)

**Table:**

C1—C6	1.375 (4)	C10—C15	1.398 (4)
C1—C2	1.380 (4)	C11—C12	1.405 (4)
C1—S1	1.758 (3)	C11—C17	1.492 (4)
C2—C3	1.377 (5)	C12—C13	1.364 (5)
C2—H2	0.9300	C12—H12	0.9300
C3—C4	1.378 (6)	C13—C14	1.369 (5)
C3—H3	0.9300	C13—H13	0.9300
C4—C5	1.370 (6)	C14—C15	1.378 (4)
C4—C16	1.516 (6)	C14—H14	0.9300
C5—C6	1.376 (5)	C15—H15	0.9300
C5—H5	0.9300	C16—H16A	0.9600
C6—H6	0.9300	C16—H16B	0.9600
C7—N1	1.440 (4)	C16—H16C	0.9600
C7—C8	1.518 (4)	C17—H17A	0.9600
C7—H7A	0.9700	C17—H17B	0.9600
C7—H7B	0.9700	C17—H17C	0.9600
C8—O3	1.233 (3)	N1—S1	1.604 (3)
C8—N2	1.331 (3)	N1—H1N	0.838 (17)
C9—N3	1.270 (3)	N2—N3	1.384 (3)
C9—C10	1.470 (4)	N2—H2N	0.858 (17)
C9—H9	0.9300	O1—S1	1.422 (2)
C10—C11	1.397 (4)	O2—S1	1.431 (2)
			
C6—C1—C2	119.9 (3)	C13—C12—H12	119.3
C6—C1—S1	120.0 (2)	C11—C12—H12	119.3
C2—C1—S1	120.0 (3)	C12—C13—C14	120.9 (3)
C3—C2—C1	119.1 (4)	C12—C13—H13	119.5
C3—C2—H2	120.4	C14—C13—H13	119.5
C1—C2—H2	120.4	C13—C14—C15	119.0 (3)
C2—C3—C4	121.6 (4)	C13—C14—H14	120.5
C2—C3—H3	119.2	C15—C14—H14	120.5
C4—C3—H3	119.2	C14—C15—C10	121.4 (3)
C5—C4—C3	118.2 (4)	C14—C15—H15	119.3
C5—C4—C16	121.3 (5)	C10—C15—H15	119.3
C3—C4—C16	120.5 (5)	C4—C16—H16A	109.5
C4—C5—C6	121.2 (4)	C4—C16—H16B	109.5
C4—C5—H5	119.4	H16A—C16—H16B	109.5
C6—C5—H5	119.4	C4—C16—H16C	109.5
C1—C6—C5	119.8 (4)	H16A—C16—H16C	109.5
C1—C6—H6	120.1	H16B—C16—H16C	109.5
C5—C6—H6	120.1	C11—C17—H17A	109.5
N1—C7—C8	113.2 (2)	C11—C17—H17B	109.5
N1—C7—H7A	108.9	H17A—C17—H17B	109.5
C8—C7—H7A	108.9	C11—C17—H17C	109.5
N1—C7—H7B	108.9	H17A—C17—H17C	109.5
C8—C7—H7B	108.9	H17B—C17—H17C	109.5
H7A—C7—H7B	107.7	C7—N1—S1	122.9 (2)
O3—C8—N2	121.5 (2)	C7—N1—H1N	122 (2)
O3—C8—C7	122.1 (2)	S1—N1—H1N	115 (2)
N2—C8—C7	116.5 (2)	C8—N2—N3	119.6 (2)
N3—C9—C10	119.4 (2)	C8—N2—H2N	120 (2)
N3—C9—H9	120.3	N3—N2—H2N	120 (2)
C10—C9—H9	120.3	C9—N3—N2	117.0 (2)
C11—C10—C15	119.3 (3)	O1—S1—O2	120.42 (15)
C11—C10—C9	121.8 (2)	O1—S1—N1	106.98 (15)
C15—C10—C9	118.9 (2)	O2—S1—N1	107.14 (15)
C10—C11—C12	117.9 (3)	O1—S1—C1	108.74 (15)
C10—C11—C17	123.0 (3)	O2—S1—C1	107.04 (15)
C12—C11—C17	119.1 (3)	N1—S1—C1	105.61 (15)
C13—C12—C11	121.4 (3)		
			
C6—C1—C2—C3	1.8 (5)	C11—C12—C13—C14	0.0 (6)
S1—C1—C2—C3	−174.6 (3)	C12—C13—C14—C15	−0.7 (6)
C1—C2—C3—C4	0.1 (6)	C13—C14—C15—C10	1.0 (5)
C2—C3—C4—C5	−2.8 (6)	C11—C10—C15—C14	−0.5 (5)
C2—C3—C4—C16	177.9 (4)	C9—C10—C15—C14	178.5 (3)
C3—C4—C5—C6	3.7 (6)	C8—C7—N1—S1	−84.6 (3)
C16—C4—C5—C6	−177.0 (4)	O3—C8—N2—N3	179.7 (3)
C2—C1—C6—C5	−0.9 (5)	C7—C8—N2—N3	−1.5 (4)
S1—C1—C6—C5	175.5 (3)	C10—C9—N3—N2	−179.4 (2)
C4—C5—C6—C1	−1.9 (6)	C8—N2—N3—C9	−177.1 (3)
N1—C7—C8—O3	−15.8 (4)	C7—N1—S1—O1	16.2 (3)
N1—C7—C8—N2	165.4 (3)	C7—N1—S1—O2	146.6 (2)
N3—C9—C10—C11	−176.8 (3)	C7—N1—S1—C1	−99.6 (3)
N3—C9—C10—C15	4.3 (4)	C6—C1—S1—O1	148.8 (3)
C15—C10—C11—C12	−0.3 (4)	C2—C1—S1—O1	−34.8 (3)
C9—C10—C11—C12	−179.3 (3)	C6—C1—S1—O2	17.2 (3)
C15—C10—C11—C17	178.8 (3)	C2—C1—S1—O2	−166.4 (3)
C9—C10—C11—C17	−0.2 (5)	C6—C1—S1—N1	−96.7 (3)
C10—C11—C12—C13	0.5 (5)	C2—C1—S1—N1	79.7 (3)
C17—C11—C12—C13	−178.6 (3)		

### (E)-N-{2-[2-(2-Methylbenzylidene)hydrazinyl]-2-oxoethyl}-4-methylbenzenesulfonamide (II) Hydrogen-bond geometry (Å, º)

**Table:**

*D*—H···*A*	*D*—H	H···*A*	*D*···*A*	*D*—H···*A*
N1—H1*N*···O3^i^	0.84 (2)	2.03 (2)	2.845 (3)	166 (3)
N2—H2*N*···O3^ii^	0.86 (2)	2.05 (2)	2.898 (3)	170 (3)

Symmetry codes: (i) −*x*, −*y*+1, −*z*+2; (ii) −*x*−1, −*y*+1, −*z*+2.

### (E)-N-{2-[2-(2-Nitrobenzylidene)hydrazinyl]-2-oxoethyl}-4-methylbenzenesulfonamide (III) Crystal data

**Table:**

C_16_H_16_N_4_O_5_S	*Z* = 2
*M**_r_* = 376.39	*F*(000) = 392
Triclinic, *P*1	*D*_x_ = 1.446 Mg m^−^^3^
*a* = 8.006 (1) Å	Mo *K*α radiation, λ = 0.71073 Å
*b* = 10.229 (1) Å	Cell parameters from 1476 reflections
*c* = 11.181 (2) Å	θ = 2.7–27.9°
α = 83.76 (1)°	µ = 0.22 mm^−^^1^
β = 72.86 (1)°	*T* = 293 K
γ = 82.13 (1)°	Rod, light yellow
*V* = 864.5 (2) Å^3^	0.48 × 0.48 × 0.28 mm

### (E)-N-{2-[2-(2-Nitrobenzylidene)hydrazinyl]-2-oxoethyl}-4-methylbenzenesulfonamide (III) Data collection

**Table:**

Oxford Diffraction Xcalibur diffractometer with Sapphire CCD detector	2511 reflections with *I* > 2σ(*I*)
Radiation source: Enhance (Mo) X-ray Source	*R*_int_ = 0.020
Rotation method data acquisition using ω scans	θ_max_ = 26.4°, θ_min_ = 2.7°
Absorption correction: multi-scan CrysAlis RED (Oxford Diffraction, 2009)	*h* = −10→10
*T*_min_ = 0.900, *T*_max_ = 0.940	*k* = −8→12
5980 measured reflections	*l* = −13→13
3524 independent reflections	

### (E)-N-{2-[2-(2-Nitrobenzylidene)hydrazinyl]-2-oxoethyl}-4-methylbenzenesulfonamide (III) Refinement

**Table:**

Refinement on *F*^2^	10 restraints
Least-squares matrix: full	Hydrogen site location: mixed
*R*[*F*^2^ > 2σ(*F*^2^)] = 0.057	H atoms treated by a mixture of independent and constrained refinement
*wR*(*F*^2^) = 0.131	*w* = 1/[σ^2^(*F*_o_^2^) + (0.0346*P*)^2^ + 0.6348*P*] where *P* = (*F*_o_^2^ + 2*F*_c_^2^)/3
*S* = 1.16	(Δ/σ)_max_ < 0.001
3524 reflections	Δρ_max_ = 0.31 e Å^−^^3^
252 parameters	Δρ_min_ = −0.30 e Å^−^^3^

### (E)-N-{2-[2-(2-Nitrobenzylidene)hydrazinyl]-2-oxoethyl}-4-methylbenzenesulfonamide (III) Special details

**Table:**

Geometry. All esds (except the esd in the dihedral angle between two l.s. planes) are estimated using the full covariance matrix. The cell esds are taken into account individually in the estimation of esds in distances, angles and torsion angles; correlations between esds in cell parameters are only used when they are defined by crystal symmetry. An approximate (isotropic) treatment of cell esds is used for estimating esds involving l.s. planes.

### (E)-N-{2-[2-(2-Nitrobenzylidene)hydrazinyl]-2-oxoethyl}-4-methylbenzenesulfonamide (III) Fractional atomic coordinates and isotropic or equivalent isotropic displacement parameters (Å^2^)

**Table:**

	*x*	*y*	*z*	*U*_iso_*/*U*_eq_	Occ. (<1)
C1	0.1583 (4)	0.0902 (3)	0.3328 (3)	0.0432 (7)	
C2	0.0013 (4)	0.0489 (3)	0.3328 (3)	0.0515 (7)	
H2	0.0012	−0.0291	0.2972	0.062*	
C3	−0.1551 (4)	0.1232 (4)	0.3853 (3)	0.0646 (9)	
H3	−0.2603	0.0939	0.3857	0.078*	
C4	−0.1592 (5)	0.2390 (4)	0.4367 (3)	0.0679 (9)	
C5	−0.0013 (5)	0.2782 (3)	0.4388 (3)	0.0695 (9)	
H5	−0.0021	0.3558	0.4754	0.083*	
C6	0.1581 (5)	0.2045 (3)	0.3877 (3)	0.0603 (8)	
H6	0.2630	0.2317	0.3903	0.072*	
C7	0.5648 (3)	0.1686 (3)	0.1001 (3)	0.0490 (7)	
H7A	0.5499	0.2132	0.1754	0.059*	
H7B	0.5508	0.2354	0.0342	0.059*	
C8	0.7488 (3)	0.0959 (2)	0.0614 (2)	0.0364 (6)	
C9	0.9667 (3)	0.3431 (2)	0.1185 (2)	0.0380 (6)	
H9A	1.0802	0.2998	0.1013	0.046*	
C10	0.9269 (3)	0.4777 (2)	0.1623 (2)	0.0362 (6)	
C11	1.0452 (3)	0.5481 (3)	0.1918 (3)	0.0416 (6)	
C12	0.9983 (4)	0.6727 (3)	0.2364 (3)	0.0543 (8)	
H12	1.0797	0.7158	0.2574	0.065*	
C13	0.8299 (4)	0.7322 (3)	0.2493 (3)	0.0584 (9)	
H13	0.7974	0.8166	0.2778	0.070*	
C14	0.7102 (4)	0.6671 (3)	0.2202 (3)	0.0544 (8)	
H14	0.5966	0.7079	0.2283	0.065*	
C15	0.7571 (4)	0.5416 (3)	0.1790 (3)	0.0446 (7)	
H15	0.6730	0.4981	0.1618	0.054*	
C16	−0.3304 (6)	0.3242 (5)	0.4884 (4)	0.1112 (18)	
H16A	−0.4218	0.2687	0.5278	0.167*	
H16B	−0.3609	0.3795	0.4212	0.167*	
H16C	−0.3172	0.3787	0.5490	0.167*	
N1	0.4300 (3)	0.0805 (3)	0.1237 (2)	0.0496 (6)	
H1N	0.385 (4)	0.071 (3)	0.066 (2)	0.059*	
N2	0.8798 (3)	0.1627 (2)	0.0649 (2)	0.0386 (5)	
H2N	0.987 (2)	0.125 (3)	0.043 (3)	0.046*	
N3	0.8416 (3)	0.2882 (2)	0.1050 (2)	0.0385 (5)	
N4	1.2287 (3)	0.4938 (3)	0.1766 (3)	0.0599 (7)	
O1	0.3169 (3)	−0.12378 (19)	0.2315 (2)	0.0650 (6)	
O2	0.4818 (3)	0.0038 (2)	0.3249 (2)	0.0692 (7)	
O3	0.7787 (2)	−0.01737 (17)	0.02690 (19)	0.0447 (5)	
O4	1.3001 (3)	0.4196 (3)	0.0977 (3)	0.0919 (9)	
O5	1.2976 (6)	0.5163 (5)	0.2569 (6)	0.102 (2)	0.836 (12)
O5'	1.3314 (19)	0.5818 (18)	0.157 (3)	0.083 (9)	0.164 (12)
S1	0.35831 (9)	0.00025 (7)	0.25622 (8)	0.0478 (2)	

### (E)-N-{2-[2-(2-Nitrobenzylidene)hydrazinyl]-2-oxoethyl}-4-methylbenzenesulfonamide (III) Atomic displacement parameters (Å^2^)

**Table:**

	*U*^11^	*U*^22^	*U*^33^	*U*^12^	*U*^13^	*U*^23^
C1	0.0411 (15)	0.0425 (15)	0.0418 (16)	−0.0022 (12)	−0.0074 (12)	−0.0006 (13)
C2	0.0421 (16)	0.0555 (18)	0.0552 (19)	−0.0072 (14)	−0.0111 (14)	−0.0017 (15)
C3	0.0407 (18)	0.085 (3)	0.060 (2)	−0.0004 (17)	−0.0080 (15)	0.0025 (19)
C4	0.0641 (19)	0.078 (2)	0.0408 (18)	0.0169 (17)	0.0032 (16)	0.0048 (17)
C5	0.090 (2)	0.057 (2)	0.0508 (19)	0.0026 (18)	−0.0040 (19)	−0.0157 (16)
C6	0.060 (2)	0.061 (2)	0.058 (2)	−0.0107 (16)	−0.0077 (16)	−0.0144 (16)
C7	0.0309 (14)	0.0424 (16)	0.070 (2)	−0.0009 (12)	−0.0063 (13)	−0.0154 (14)
C8	0.0307 (13)	0.0356 (14)	0.0419 (15)	−0.0021 (11)	−0.0087 (11)	−0.0046 (12)
C9	0.0307 (13)	0.0351 (14)	0.0472 (16)	−0.0013 (11)	−0.0093 (12)	−0.0056 (12)
C10	0.0356 (14)	0.0303 (13)	0.0410 (15)	−0.0035 (11)	−0.0086 (11)	−0.0022 (11)
C11	0.0374 (15)	0.0353 (14)	0.0529 (17)	−0.0041 (11)	−0.0140 (13)	−0.0027 (13)
C12	0.0551 (19)	0.0406 (16)	0.072 (2)	−0.0115 (14)	−0.0204 (16)	−0.0126 (15)
C13	0.060 (2)	0.0361 (16)	0.076 (2)	0.0019 (14)	−0.0137 (17)	−0.0178 (15)
C14	0.0436 (17)	0.0430 (17)	0.071 (2)	0.0059 (13)	−0.0108 (15)	−0.0110 (15)
C15	0.0351 (14)	0.0405 (15)	0.0577 (18)	−0.0037 (12)	−0.0110 (13)	−0.0083 (13)
C16	0.090 (3)	0.122 (4)	0.078 (3)	0.047 (3)	0.013 (2)	0.001 (3)
N1	0.0324 (13)	0.0623 (16)	0.0556 (16)	−0.0106 (11)	−0.0081 (11)	−0.0166 (13)
N2	0.0278 (11)	0.0345 (12)	0.0523 (14)	0.0000 (9)	−0.0079 (10)	−0.0115 (10)
N3	0.0342 (12)	0.0303 (11)	0.0497 (14)	−0.0017 (9)	−0.0090 (10)	−0.0077 (10)
N4	0.0433 (15)	0.0456 (15)	0.100 (2)	−0.0046 (12)	−0.0302 (16)	−0.0160 (15)
O1	0.0550 (13)	0.0384 (11)	0.1000 (18)	−0.0020 (10)	−0.0181 (13)	−0.0126 (11)
O2	0.0496 (13)	0.0867 (17)	0.0790 (16)	0.0004 (12)	−0.0331 (12)	−0.0076 (13)
O3	0.0331 (10)	0.0361 (10)	0.0667 (13)	0.0007 (8)	−0.0142 (9)	−0.0177 (9)
O4	0.0441 (14)	0.0826 (18)	0.153 (3)	0.0129 (13)	−0.0299 (16)	−0.0465 (19)
O5	0.077 (3)	0.109 (4)	0.154 (5)	0.002 (2)	−0.076 (3)	−0.042 (4)
O5'	0.046 (8)	0.069 (11)	0.139 (19)	−0.014 (7)	−0.027 (9)	−0.020 (11)
S1	0.0350 (4)	0.0450 (4)	0.0644 (5)	−0.0004 (3)	−0.0156 (3)	−0.0093 (3)

### (E)-N-{2-[2-(2-Nitrobenzylidene)hydrazinyl]-2-oxoethyl}-4-methylbenzenesulfonamide (III) Geometric parameters (Å, º)

**Table:**

C1—C6	1.377 (4)	C10—C11	1.395 (4)
C1—C2	1.380 (4)	C11—C12	1.383 (4)
C1—S1	1.762 (3)	C11—N4	1.464 (4)
C2—C3	1.376 (4)	C12—C13	1.373 (4)
C2—H2	0.9300	C12—H12	0.9300
C3—C4	1.364 (5)	C13—C14	1.369 (4)
C3—H3	0.9300	C13—H13	0.9300
C4—C5	1.385 (5)	C14—C15	1.377 (4)
C4—C16	1.513 (5)	C14—H14	0.9300
C5—C6	1.387 (5)	C15—H15	0.9300
C5—H5	0.9300	C16—H16A	0.9600
C6—H6	0.9300	C16—H16B	0.9600
C7—N1	1.446 (4)	C16—H16C	0.9600
C7—C8	1.518 (3)	N1—S1	1.600 (3)
C7—H7A	0.9700	N1—H1N	0.845 (17)
C7—H7B	0.9700	N2—N3	1.371 (3)
C8—O3	1.232 (3)	N2—H2N	0.865 (17)
C8—N2	1.340 (3)	N4—O4	1.188 (3)
C9—N3	1.266 (3)	N4—O5	1.239 (4)
C9—C10	1.474 (3)	N4—O5'	1.260 (13)
C9—H9A	0.9300	O1—S1	1.429 (2)
C10—C15	1.394 (4)	O2—S1	1.426 (2)
			
C6—C1—C2	119.9 (3)	C10—C11—N4	121.6 (2)
C6—C1—S1	120.4 (2)	C13—C12—C11	119.2 (3)
C2—C1—S1	119.6 (2)	C13—C12—H12	120.4
C3—C2—C1	120.0 (3)	C11—C12—H12	120.4
C3—C2—H2	120.0	C14—C13—C12	120.0 (3)
C1—C2—H2	120.0	C14—C13—H13	120.0
C4—C3—C2	121.4 (3)	C12—C13—H13	120.0
C4—C3—H3	119.3	C13—C14—C15	120.3 (3)
C2—C3—H3	119.3	C13—C14—H14	119.8
C3—C4—C5	118.2 (3)	C15—C14—H14	119.8
C3—C4—C16	121.4 (4)	C14—C15—C10	122.0 (3)
C5—C4—C16	120.4 (4)	C14—C15—H15	119.0
C4—C5—C6	121.5 (3)	C10—C15—H15	119.0
C4—C5—H5	119.2	C4—C16—H16A	109.5
C6—C5—H5	119.2	C4—C16—H16B	109.5
C1—C6—C5	118.9 (3)	H16A—C16—H16B	109.5
C1—C6—H6	120.6	C4—C16—H16C	109.5
C5—C6—H6	120.6	H16A—C16—H16C	109.5
N1—C7—C8	112.3 (2)	H16B—C16—H16C	109.5
N1—C7—H7A	109.1	C7—N1—S1	122.9 (2)
C8—C7—H7A	109.1	C7—N1—H1N	119 (2)
N1—C7—H7B	109.1	S1—N1—H1N	118 (2)
C8—C7—H7B	109.1	C8—N2—N3	119.4 (2)
H7A—C7—H7B	107.9	C8—N2—H2N	119.2 (19)
O3—C8—N2	121.0 (2)	N3—N2—H2N	121.4 (19)
O3—C8—C7	122.7 (2)	C9—N3—N2	117.5 (2)
N2—C8—C7	116.3 (2)	O4—N4—C11	120.1 (3)
N3—C9—C10	118.0 (2)	O5—N4—C11	117.5 (3)
N3—C9—H9A	121.0	O5'—N4—C11	113.1 (9)
C10—C9—H9A	121.0	O2—S1—O1	120.10 (15)
C15—C10—C11	115.8 (2)	O2—S1—N1	106.98 (14)
C15—C10—C9	118.8 (2)	O1—S1—N1	107.32 (14)
C11—C10—C9	125.4 (2)	O2—S1—C1	108.77 (14)
C12—C11—C10	122.7 (3)	O1—S1—C1	107.00 (13)
C12—C11—N4	115.7 (3)	N1—S1—C1	105.82 (13)
			
C6—C1—C2—C3	1.2 (5)	C11—C10—C15—C14	1.0 (4)
S1—C1—C2—C3	−175.8 (2)	C9—C10—C15—C14	179.7 (3)
C1—C2—C3—C4	0.8 (5)	C8—C7—N1—S1	85.4 (3)
C2—C3—C4—C5	−2.2 (5)	O3—C8—N2—N3	−179.4 (2)
C2—C3—C4—C16	176.8 (3)	C7—C8—N2—N3	1.6 (4)
C3—C4—C5—C6	1.5 (5)	C10—C9—N3—N2	−179.7 (2)
C16—C4—C5—C6	−177.4 (3)	C8—N2—N3—C9	173.4 (2)
C2—C1—C6—C5	−1.9 (5)	C12—C11—N4—O4	−149.9 (3)
S1—C1—C6—C5	175.2 (3)	C10—C11—N4—O4	29.8 (5)
C4—C5—C6—C1	0.5 (5)	C12—C11—N4—O5	38.0 (5)
N1—C7—C8—O3	15.4 (4)	C10—C11—N4—O5	−142.3 (5)
N1—C7—C8—N2	−165.5 (2)	C12—C11—N4—O5'	−27.0 (16)
N3—C9—C10—C15	−4.1 (4)	C10—C11—N4—O5'	152.6 (16)
N3—C9—C10—C11	174.5 (3)	C7—N1—S1—O2	−15.9 (3)
C15—C10—C11—C12	0.7 (4)	C7—N1—S1—O1	−146.1 (2)
C9—C10—C11—C12	−177.9 (3)	C7—N1—S1—C1	99.9 (2)
C15—C10—C11—N4	−178.9 (3)	C6—C1—S1—O2	37.8 (3)
C9—C10—C11—N4	2.5 (4)	C2—C1—S1—O2	−145.2 (2)
C10—C11—C12—C13	−1.8 (5)	C6—C1—S1—O1	168.9 (2)
N4—C11—C12—C13	177.9 (3)	C2—C1—S1—O1	−14.0 (3)
C11—C12—C13—C14	1.1 (5)	C6—C1—S1—N1	−76.8 (3)
C12—C13—C14—C15	0.6 (5)	C2—C1—S1—N1	100.2 (2)
C13—C14—C15—C10	−1.7 (5)		

### (E)-N-{2-[2-(2-Nitrobenzylidene)hydrazinyl]-2-oxoethyl}-4-methylbenzenesulfonamide (III) Hydrogen-bond geometry (Å, º)

**Table:**

*D*—H···*A*	*D*—H	H···*A*	*D*···*A*	*D*—H···*A*
N1—H1*N*···O3^i^	0.85 (2)	2.06 (2)	2.869 (3)	161 (3)
N2—H2*N*···O3^ii^	0.87 (2)	2.03 (2)	2.881 (3)	170 (3)
C7—H7*B*···O4^iii^	0.97	2.55	3.100 (4)	116
C9—H9*A*···O4	0.93	2.27	2.821 (4)	118
C16—H16*C*···O5^iv^	0.96	2.58	3.525 (6)	168

Symmetry codes: (i) −*x*+1, −*y*, −*z*; (ii) −*x*+2, −*y*, −*z*; (iii) *x*−1, *y*, *z*; (iv) −*x*+1, −*y*+1, −*z*+1.
